# Employee innovative behavior and workplace wellbeing: Leader support for innovation and coworker ostracism as mediators

**DOI:** 10.3389/fpsyg.2022.1014195

**Published:** 2022-11-29

**Authors:** Hui Wang, Xueshuang Chen, Hui Wang, Mingxing Xie

**Affiliations:** ^1^Business School, Xiangtan University, Xiangtan, China; ^2^School of Public Administration, Xiangtan University, Xiangtan, China; ^3^School of Humanity, Shanghai University of Finance and Economics, Shanghai, China

**Keywords:** employee innovative behavior, workplace wellbeing, coworker ostracism, leader support for innovation, dual mediating model

## Abstract

**Introduction:**

Most previous studies focused on the antecedents of employee innovative behavior but rarely examined the outcomes of employee innovative behavior. Moreover, previous studies ignored the relationship between employee innovative behavior and workplace wellbeing. Based on social comparison theory and social exchange theory, this study introduces coworker ostracism and leader support for innovation as mediating variables to explore the “double–edged sword” effect of employee innovative behavior on workplace wellbeing.

**Methods:**

Based on a sample of 319 employees from Chinese companies, this study used SPSS 26.0 and MPLUS 8.3 to examine the hypotheses.

**Results:**

Empirical results demonstrate that (a) employee innovative behavior is directly and positively related to workplace wellbeing, (b) employee innovative behavior is indirectly and positively related to workplace wellbeing through leader support for innovation, and (c) the negative association between employee innovative behavior and workplace wellbeing via coworker ostracism is unsupported.

**Discussion:**

The findings of this study enrich the literature by exploring the double-edged sword effect of employee innovative behavior on workplace wellbeing. The practical implications of this study are that leaders in organizations should give employees innovation support.

## Introduction

Employee innovative behavior refers to a complete process in the workplace, in which individuals generate, promote, and implement new ideas (Scott and Bruce, 1994). Research on employee innovative behavior proliferated at an increasing rate in the past several decades (Kang et al., 2016; Eva et al., 2019; Tian et al., 2020; Wang H. et al., 2021). Employee innovative behavior is generally believed to be an important source of organizational competitive advantage, which is conducive to the development of an organization (e.g., Anderson et al., 2014; Shin et al., 2017; Eva et al., 2019). Thus, most studies focused on the antecedents of employee innovative behavior to explore how to facilitate such behavior but rarely examined its outcomes. In the literature on the outcomes of employee innovative behavior, most studies focused on the benefits of such behavior to individuals or organizations, and recently, the dark side of employee innovative behavior or creativity has been gaining attention (Janssen, 2003; Janssen et al., 2004; Aryee et al., 2012; Harari et al., 2016; Kim and Koo, 2017; Hammond et al., 2019; Ng and Wang, 2019; Nguyen and Le, 2019; Breidenthal et al., 2020; Coad et al., 2021; Dadaboyev et al., 2021). For example, Aryee et al. (2012) and Kim and Koo (2017) proved the existence of a positive correlation between employee innovative behavior and job performance. Ng and Wang (2019) found that employee innovative behavior has potential costs, which may cause psychological disengagement difficulties, and an important partner effect, which may cause stress among colleagues. Breidenthal et al. (2020) also found that a relatively high level of creativity may cause coworker envy, which can lead to coworker ostracism. Although previous studies have explored the possible positive or negative effects of employees’ innovative behavior, no studies have integrated the analysis of the two different effects. Janssen et al. (2004) proposed a theoretical model to summarize the positive outcomes (e.g., improved performance, positive work attitude, constructive conflict, and workplace wellbeing) and negative outcomes (e.g., performance reduction, negative work attitude, destructive conflict, and work stress) of employee innovative behavior. Janssen et al. (2004) further suggested that researchers should develop models to explore the positive and negative outcomes of employee innovative behavior. Therefore, this study will respond to this call. In addition, with the advent of the digital economy era, social competition and work pressure increased, and workplace wellbeing attracted considerable attention from organizations (Salas-Vallina et al., 2017; Sorribes et al., 2021). Workplace wellbeing is considered to be beneficial to enterprises for retaining talents, creating a satisfactory work atmosphere, and promoting their sustainable development (Salas-Vallina et al., 2017; Nangoy et al., 2019). However, the relationship between employee innovative behavior and employee wellbeing has been largely ignored. Mustafa and Ramos (2018) proposed a conceptual model exploring how to mitigate the negative impact of employee creativity on wellbeing; however, they did not explore the mediating mechanisms nor did they conduct empirical tests. Furthermore, innovative behavior differs from individual creativity in that creativity is particularly concerned with coming up with novel ideas or solutions, whereas, innovative behavior further involves application-oriented components (Shalley et al., 2004; Hammond et al., 2011). Therefore, in the context of highly valued innovation and workplace wellbeing, examining the mechanism of how employee innovative behavior impact workplace wellbeing is of considerable significance. To fill this gap, the first objective of this research is to explore the direct relationship between employee innovative behavior and workplace wellbeing. In line with the call of Janssen et al. (2004), the second objective of our research is to explore the indirect positive effect and negative effect of employee innovative behavior on employees’ workplace wellbeing.

To reveal the relationship between employee innovative behavior and workplace wellbeing, drawing on social comparison theory and social exchange theory, this study introduces coworker ostracism and leader support for innovation as mediating variables to explore the bright side and dark side of the effect of employee innovative behavior on employees’ workplace wellbeing. This study chooses the two mediating variables for the following two reasons. First, coworkers and leaders play a vital part in the process of employees’ innovative behavior (Chiaburu and Harrison, 2008; Sijbom et al., 2015a,b). Second, employees, coworkers, and leaders belong to an organizational ecosystem (Neves and Cunha, 2017), where they interact frequently, spend a significant amount of their time at work, and are bound to influence one another to a certain extent. Specifically, this study argues that coworker ostracism is an important mediating variable between employee innovative behavior and workplace wellbeing. Because in modern society, where innovation is encouraged and competition is fierce, “shooting the top bird” has become one of the most common phenomena in the workplace. According to social comparison theory, members of the same team tend to compare themselves with their coworkers to determine their status in the organization. Therefore, employees’ outstanding innovative performance may cause their coworkers to reject them, which can adversely affect their wellbeing. Breidenthal et al. (2020) confirmed the dark side of creativity, that is, when employees demonstrate high creativity, they may cause jealousy and experience ostracism from coworkers, which may negatively affect their wellbeing. In addition, this study considers leader support for innovation as an important mediating variable between employee innovative behavior and workplace wellbeing. In a power hierarchy, employees rely on the leader for the information and support necessary to further develop after they implement innovative behaviors. A leader is a crucial party for employees to implement innovative behaviors (Kanter, 1988). Social exchange theory holds that individuals maintain an exchange relationship with others based on the principle of mutual benefit. This reciprocity principle, which is emphasized in social exchange theory, promotes the emergence of exchange. Employee innovative behavior is beneficial for not only promoting organizational performance but also improving the competitiveness of the organization. Consequently, according to social exchange theory, employees who engage in considerable innovative behavior are likely to receive innovation support from their supervisors, which can enhance their wellbeing. Furthermore, a leader has absolute power and status and is bound to exert a certain influence on the members of his/her team. Accordingly, the leader can use his/her power to minimize phenomenon such as exclusion by coworkers, which is not conducive to the development of the team. Therefore, this study suggests that leader support for innovation may also have an impact on coworker ostracism.

Overall, this study integrates social comparison theory and social exchange theory to construct a serial mediation model of the influence of employee innovative behavior on workplace wellbeing, which uses coworker ostracism and leader support for innovation as mediating variables. This study may have several contributions. First, this study discusses the direct relationship between employee innovative behavior and workplace wellbeing, which can provide a new perspective on the adoption of employee innovative behavior as an antecedent variable, and expands research on the outcomes of employee innovative behavior. Second, based on social comparison theory and social exchange theory, this study introduces coworker ostracism and leader support for innovation as two mediating variables to discuss the indirect positive effect and negative effect of employee innovative behavior on employee workplace wellbeing, which can enrich the literature on the relationship between the two factors. Previous literature has paid limited attention to the dark side of employee innovative behavior. Drawing on social comparison theory, this study takes step to explore the negative impact of employee innovation behavior on employee wellbeing. More importantly, this study integrates, for the first time, the double-edged effect of employee innovative behavior on workplace wellbeing through the negative effect of coworker ostracism and the positive effect of leader support for innovation. Third, this study explores the chain-mediating path of “leader support for innovation–coworker ostracism” between employee innovative behavior and workplace wellbeing, further revealing the mechanism of the effect of employee innovative behavior on workplace wellbeing.

## Theoretical background and hypotheses

### Employee innovative behavior and workplace wellbeing

Employee innovative behavior refers to employees’ creation of novel ideas or methods and their implementation in practice in the process of work. Employee innovative behavior involves three stages: generating innovative ideas, seeking coalitions of supporters, and implementing the innovative ideas in practice (Scott and Bruce, 1994). Workplace wellbeing refers to employees’ positive psychological state and experience in the process of fulfilling their self-realization goals and is an important indicator of their mental health, which roughly includes three perspectives: subjective wellbeing, psychological wellbeing, and integrated wellbeing (Diener et al., 1985; Page and Vella-Brodrick, 2008). Terkel (1974) argued that work is the process of searching for bread and meaning every day as well as for cash and recognition. On the one hand, employee innovative behavior can generate high compensation and income to meet employees’ material needs. On the other hand, employee innovative behavior may generate increased value for an enterprise and the society and meet employees’ self-realization needs, thereby improving their workplace wellbeing. Accordingly, this study deduces that employee innovative behavior is directly and positively related to workplace wellbeing.

First, employees who exhibit considerable innovative behavior may be rewarded financially. Specifically, innovation may have corresponding rewards and meet the material needs of employees, thereby improving their workplace wellbeing. Studies confirmed the positive impact of income on happiness. Kollamparambil (2019) examined four dynamic data of national income in South Africa and found that income can determine the level of happiness. Rijnks et al. (2019) observed that absolute income and relative income can determine personal happiness. Second, innovative behavior means that employees’ abilities and skills are improved in the process of continuous innovation, and corporate value and social value are enhanced to meet the spiritual needs of employees for self-improvement and self-value realization, thereby improving their workplace wellbeing. The constant realization of inner goals can help individuals maintain a stable sense of wellbeing (Schmuck et al., 2000). Page and Vella-Brodrick (2008) determined that self-improvement based on strength can reliably improve happiness. Meanwhile, Duan et al. (2020) reported that psychological meaning and perceived social value are positively correlated with workplace wellbeing. Moreover, in the context of Chinese collectivist culture, people pay considerable attention to their social value. Therefore, employees’ innovative behavior can not only generate value for the enterprise and society but also enhance their happiness. Finally, according to the hierarchy of needs theory, human beings have five levels of needs: physiological, safety, social, respect, and self-realization, which transition from material to spiritual needs. Innovation, as a risky and valuable activity, is the affirmation of the innovative abilities of employees. Moreover, innovation can increase economic rewards for employees, generate substantial economic value for enterprises and society, and meet the material and spiritual needs of employees. Based on the above discussion, this study proposes the following hypothesis:

*Hypothesis 1: Employee innovative behavior is directly and positively associated with workplace wellbeing.*

### Mediating role of coworker ostracism in the relationship between employee innovative behavior and workplace wellbeing

Coworker ostracism is defined as the subjective feeling of being ignored, avoided, or excluded by coworkers in the workplace (Ferris et al., 2008). Rejection by coworkers in the workplace can lead to unpleasant and painful experiences for employees (Zhang and Shi, 2017). Drawing upon social comparison theory, individuals have an inherent drive to evaluate their abilities and perspectives, especially when assessment criteria are not defined, and they will attempt to compare themselves to others who are close (Festinger, 1954), such as coworkers. Coworkers have been considered particularly likely referents to be used in the workplace, especially when assessing performance in innovative activities (Mumford, 1983). Specifically, the successful performance of an employee (e.g., innovative behavior) triggers negative upward comparisons with coworkers, and such unfavorable comparisons with peers can lead to increased coworker envy and coworker ostracism (Breidenthal et al., 2020; Dadaboyev et al., 2021), thus reducing employees’ workplace wellbeing.

On the one hand, employee innovative behavior has a correction effect on coworker ostracism. Employee innovative behavior is a type of breakthrough and change in existing situations or working conditions. Thus, coworkers may face the consequences of passively accepting the reform of the work content or work model brought about by other employees’ innovation (Cheng and Hong, 2017), such as job crafting. However, studies showed that individuals prefer to maintain the *status quo* and stick to their routines rather than change (Van Dam et al., 2008; Hon et al., 2014; Röth and Spieth, 2019; Kashan et al., 2022). Coworkers may not accept the changes brought about by innovative behavior, because such changes may create increased work requirements (Janssen, 2003). From this point of view, employee innovative behavior may lead to coworker ostracism. Meanwhile, from the perspective of social comparison, employee innovative behaviors are prone to generate social comparison, unlike intra-role behaviors, which are specified in role regulations and recognized by formal reward systems (Dadaboyev et al., 2021). In this case, members in the same team tend to compare themselves with their coworkers to determine their own attributes, and coworkers engaging in considerable innovative behavior are equivalent to setting a good example for the team. By contrast, coworkers who are set in their ways and do not innovate will seem conservative and inactive, which can lead to lowered self-evaluations (Buunk and Gibbons, 2007). Thus, to mitigate the threat of contrast effects due to upward comparisons, individuals may motivate defensive ostracism (Liu et al., 2019; Henle et al., 2022). Specifically, employees who exhibit more innovative behavior compared with their peers are perceived to be outliers, which may cause their exclusion from the team’s “one of us” classification system (Breidenthal et al., 2020). Moreover, when an employee engages in considerable innovative behavior, he/she will utilize substantial organizational innovation resources and thus may reduce the resources available to his/her coworkers (Graen and Uhl-Bien, 1995; Baer, 2012; Campbell et al., 2017), thereby resulting in coworker ostracism. Therefore, from the perspective of the social comparison mechanism and resource preservation, employee innovative behavior is a breakthrough in the current work balance and interpersonal relationship, which may lead to coworker ostracism.

On the other hand, coworker ostracism is associated with low levels of workplace wellbeing. Belongingness is a fundamental social need of humans, and human beings are born with the need to establish and maintain lasting and positive interpersonal relationships. If this basic need is not satisfied, then an individual may experience various negative effects, which may lead to psychological or behavioral disorders. A large number of empirical studies showed that coworker ostracism is associated with a variety of negative outcomes, including reduced voice behavior (Wen et al., 2018; Jahanzeb and Newell, 2020), increased stress (Sarfraz et al., 2019), increased job burnouts, and reduced OBSE, as well as organizational identification (Shafique et al., 2020). Coworker ostracism may make individuals feel that they are not accepted by the group and have no sense of belonging to the group (Janssen et al., 2004; Williams, 2007). Thus, they may face unpleasant experiences and perceive reduced workplace wellbeing. To sum up, this study holds that employee innovative behavior is positively related to coworker ostracism, whereas, coworker ostracism is negatively related to workplace wellbeing. Thus, this study proposes the following hypothesis:

*Hypothesis 2: Coworker ostracism plays a mediating role in the relationship between employee innovative behavior and workplace wellbeing. In other words, employees’ innovative behavior is indirectly and negatively related to their workplace wellbeing through coworker ostracism.*

### Mediating role of leader support for innovation in the relationship between employee innovative behavior and workplace wellbeing

Leader support for innovation refers to leaders advocating innovation in the workplace, encouraging employees to actively present new ideas, improving production technology or working methods, and providing corresponding support (West, 2000). Amabile et al. (1996) argued that leader support for innovation will enable leaders to set clear goals for their subordinates, actively interact with their subordinates, and support work-related innovation. Deci and Ryan (2013) proved that compared with controlling leaders, supportive leaders care more about and encourage their subordinates to actively express their ideas. According to the social exchange theory, parties engage in and maintain exchange relationships with others in anticipation of rewards (Homans, 1958; Blau, 1968), and the nature of this relationship is mutually beneficial (Emerson, 1976). Thus, when employees exhibit innovative behavior, leaders provide innovative support to employees based on the principle of reciprocity. As a result, employees see that when they are engaged in the organization, the organization likewise gives them feedback to nurture and maintain a mutually satisfying relationship, thereby enhancing employees’ workplace wellbeing.

On the one hand, employee innovative behavior may lead to leader support for innovation. Innovation emphasizes the successful implementation of innovative ideas (Amabile, 1988; Staw, 1990; Unsworth et al., 2000; Hammond et al., 2011; Montani et al., 2018), and these ideas may provide leaders not only with valuable information about emerging work-related problems but also with a creative resolution of these problems emerging in leaders’ domain of responsibility. Researches confirmed that members’ creative performance is beneficial and vital to teams, enterprises, and large social groups (Janssen et al., 2004; Yuan and Woodman, 2010; Juliao-Rossi et al., 2020). Employee innovative behavior is recognized and encouraged by leaders because it is beneficial to organization survival in the modern competitive environment. Moreover, as a type of extra-role behavior (Cheng and Hong, 2017; Coetzer et al., 2018), employee innovative behavior is beyond the scope of employees’ responsibilities. According to social exchange theory, individuals are satisfied with each other through the exchange (Homans, 1958). Employees who engage in considerable innovative behavior devote substantial amounts of time and energy and bear increased innovative risks and thus typically receive substantial support and resources from their leaders. As innovation can benefit an organization and leaders in terms of performance evaluation indicators (Eisenberger et al., 1990; Madrid et al., 2016), leaders will likely encourage and support employees who engage in considerable innovative behavior.

On the other hand, leader support for innovation can facilitate employees’ workplace wellbeing. Existing studies confirmed that leader support can significantly positively predict employees’ workplace wellbeing (Kim et al., 2018; Cohen and McKay, 2020; Hammer et al., 2021). Leader support for innovation can also improve employees’ positive emotions and stimulate their enthusiasm for work, thereby enhancing their workplace wellbeing. In addition, leader support for innovation has a positive impact on employees’ health (Hammer et al., 2013; Wang et al., 2013). Leader support for innovation means that leaders provide resource support and emotional care to their subordinates who show considerable innovative behavior (Akbari et al., 2021; Tan et al., 2021). When employees encounter difficulties in the innovation process, leaders will be understanding and will encourage them, which is conducive to reducing their insecurities and improving their workplace wellbeing. To sum up, this study proposes that employee innovative behavior is positively related to leader support for innovation, and leader support for innovation can improve employees’ workplace wellbeing. Thus, we propose the following hypothesis:

*Hypothesis 3: Leader support for innovation plays a mediating role in the relationship between employee innovative behavior and workplace wellbeing. In other words, employee innovative behavior is indirectly and positively related to workplace wellbeing through leader support for innovation.*

### Chain-mediating role of leader support for innovation and coworker ostracism in the relationship between employee innovative behavior and workplace wellbeing

In a work team, the leader and coworkers mainly constitute the interpersonal work environment. On the one hand, employees who engage in considerable innovative behavior will attract the attention of their coworkers, because such action is novel and deviates from general workplace practices and procedures. On the other hand, employees who exhibit considerable innovative behavior tend to receive increased leader support for innovation. In addition, as the power holder and resource distributor in the team, a leader will have a significant influence on the attitude and behavior of each member in the team, and his/her attitude and behavior may also directly or indirectly intervene in the process of exclusion (Rhoades and Eisenberger, 2002). Previous studies suggested that when employees and coworkers are in conflict, leaders often take on the role of a third party to reduce the negative impact of the conflict on the participants involved (Jehn and Bendersky, 2003; Peterson and Harvey, 2009). Therefore, this study suggests that leader support for innovation may offset the negative impact of coworker ostracism when employee innovative behavior has an impact on workplace wellbeing.

As for the phenomenon of coworker ostracism in the workplace, existing studies found that organizational support can alleviate the negative impact of coworker ostracism, thereby enabling employees to achieve high performance and self-worth (Scott et al., 2014; Meng, 2016). For example, Janssen and Giebels (2013) confirmed that leaders alleviated tensions and conflicts with colleagues over creative behaviors. Ali et al. (2020) found that spiritual leadership is negatively associated with workplace ostracism, both directly and indirectly *via* job social support. Therefore, leaders can relieve work pressure on employees through daily care and help and support other employees in the team to reduce their negative emotions of tension and jealousy (Lee and Duffy, 2019; Li et al., 2021). In addition, according to equity theory, leaders take steps to mitigate the effect of coworker ostracism to encourage innovative thinking. For instance, when employees engage in considerable innovative behavior, leaders will give them substantial encouragement and rewards and will tend to protect their rights and interests. Moreover, the innovation atmosphere in a team can promote cooperative behavior in innovation (Fredrickson, 2004). When employees’ innovative behavior is encouraged and supported by leaders, and when employees receive certain material and spiritual rewards, an atmosphere encouraging and supporting innovation will be formed in the organization (Su et al., 2019). Thus, employees will regard their innovative coworkers as role models instead of exhibiting jealousy or rejection. Therefore, for employees who engage in considerable innovative behavior, leaders can adopt a series of measures to reduce ostracism by coworkers to improve their workplace wellbeing. Hence, this study proposes the following hypothesis:

*Hypothesis 4: Leader support for innovation and coworker ostracism play a chain mediating role in the relationship between employee innovative behavior and workplace wellbeing. In other words, leader support for innovation is negatively related to coworker ostracism, and employee innovative behavior is indirectly related to workplace wellbeing through the chain mediating path of “leader support for innovation–coworker ostracism.”*

**FIGURE 1:**
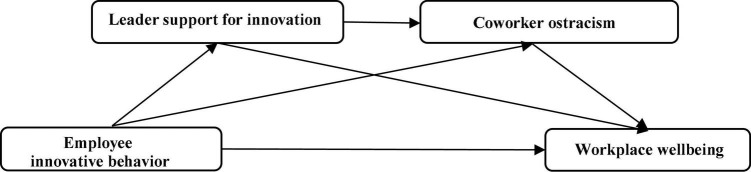
Theoretical model.

The theoretical framework is presented in Figure 1.

## Materials and methods

### Sample and procedures

In this study, the snowball sampling approach was employed to collect the company sample (Hendricks and Blanken, 1992). First, 20 companies in China were identified through MBA alumni. Second, the human resource department directors of the companies were contacted to explain the purpose of the data collection. Third, 378 employees were recruited from the 20 companies to participate in the questionnaire survey. Several days before the administration of the questionnaire survey, a private email was sent to all the participants to emphasize that the research was anonymous and only for academic research purposes and further explains the research procedure.

Podsakoff et al. (2012) suggested that multi-wave data collection for the dependent and independent constructs may be beneficial for mitigating common method variance. Following this suggestion, a two-wave data collection procedure was implemented in this study. In Time 1, the participants were required to complete a questionnaire on the independent variable (employee innovative behavior), mediating variables (coworker ostracism and leader support for innovation), and demographic variables (age, gender, education, department, and number of years employed). After a month, in time 2, the same participants were required to complete a questionnaire on the dependent variable (workplace wellbeing). To match the responses of T1 and T2, participants were asked to fill in the last four digits of their phone numbers in the questionnaire.

At time 1, 378 questionnaires were collected, and at time 2, only 343 questionnaires were collected. Among 343 questionnaires, a total of 24 questionnaires were discarded owing to missing data; patterned responses, such as alternating between the options or clicking on the midpoint; or random responses (McKibben and Silvia, 2015), thereby leaving 319 valid questionnaires, with a response rate at 84.4%. The sample description is presented in Table 1.

**TABLE 1:** Statistical characteristics of the sample.

Characteristics	Classification	Number	Ratio
Gender	Male	161	50.47%
	Female	158	49.53%
Age	18–25	98	30.72%
	25–29	113	35.42%
	30–39	94	29.47%
	40–49	13	4.08%
	≥50	1	0.31%
Education	High school or below	14	4.39%
	Junior college	37	11.60%
	Bachelor	230	72.10%
	Master or above	38	11.91%
Department	Management	82	25.71%
	Technical/R&D	114	35.74%
	Marketing	38	11.91%
	Finance	23	7.21%
	Others	62	19.44%
Working seniority	Less than 3 years	121	37.93%
	3–6 years	110	34.48%
	7–14 years	73	22.88%
	More than 15 years	15	4.70%

### Measures

The main variables in this study were employee innovative behavior, coworker ostracism, leader support for innovation, and workplace wellbeing. In addition to the control variables, each variable was measured on a five-point Likert scale ranging from 1 = “strongly disagree” to 5 = “strongly agree.” The specific application is described below.

#### Employee innovative behavior

Employee innovative behavior was measured with a six-item scale developed by Scott and Bruce (1994). The items were (1) “I search out new technologies, processes, techniques, and/or product ideas”; (2) “I often generate creative ideas”; (3) “I often promote and champion ideas to others”; (4) “I investigate and secure funds needed to implement new ideas”; (5) “I develop adequate plans and schedules for the implementation of new ideas”; and (6) “Overall, I am innovative.”

#### Coworker ostracism

Coworker ostracism was measured with the 10-item scale developed by Ferris et al. (2008). The items included (1) “I feel that my colleagues ignore me at work”; (2) “My colleagues leave the area when I enter”; (3) “My greetings are unanswered at work”; (4) “I involuntarily sit alone in a crowded lunchroom at work”; (5) “I feel that my colleagues avoid me at work”; (6) “I notice that my colleagues would not look at me at work”; (7) “I feel that my colleagues shut me out of the conversations at work”; (8) “I feel that my colleagues refuse to talk to me at work”; (9) “I feel that my colleagues treat me as if I am not there”; and (10) “My colleagues at work do not invite me or ask me if I want anything when they go out for a coffee break.”

#### Leader support for innovation

Leader support for innovation was measured with a four-item scale adapted from Amabile et al. (1996) and Vincent-Höper and Stein (2019). The items were (1) “My supervisor encourages subordinates to contribute innovative ideas or suggestions for improvement”; (2) “My supervisor advises subordinates on how to develop and implement innovative ideas in the organization”; (3) “My supervisor attempts to create satisfactory conditions for the implementation of innovative ideas, such as financial resources and flexible scheduling”; and (4) “My supervisor praises and rewards innovative behavior at work.”

#### Workplace wellbeing

Workplace wellbeing was measured with the five-item scale developed by Diener et al. (1985). The items included (1) “In most ways, I think my life is close to my ideal,” (2) “I think my life conditions are excellent,” (3) “I am satisfied with my life,” (4) “So far I have gotten the important things I want in life,” and (5) “If I could live my life over, I would change almost nothing.”

Moreover, based on previous studies, the following control variables were selected: gender (1 = male, 2 = female), age (1 = 18–25 years, 2 = 25–29 years, 3 = 30–39 years, 4 = 40–49 years, 5 = 50 years and above), education (1 = high school or below, 2 = junior college, 3 = bachelor’s degree, 4 = master’s degree or higher), department (1 = management, 2 = technical/R&D, 3 = marketing, 4 = finance, 5 = others), and working seniority (1 = less than 3 years, 2 = 3–6 years, 3 = 7–14 years, 4 = more than 15 years). As the demographic variables may have a certain correlation with the behavior performance of the employees and an impact on employee innovative behavior, they were controlled in this study.

## Data analysis and results

### Reliability and validity tests

First, SPSS 26.0 was employed to test Cronbach’s alpha of the four scales of employee innovative behavior, coworker ostracism, leader support for innovation, and workplace wellbeing (see Table 2). Cronbach’s alpha of all the variables was larger than 0.7, thereby indicating that the reliability of the questionnaire was appropriate. Second, the average variance extracted (AVE) value of most variables was larger than 0.5 (see Table 2). Though the exception is employee innovative behavior (0.478), according to previous literature, as the composite reliability (CR) of the constructs is well above the recommended level, the internal reliability of the measurement items is acceptable (Lam, 2012), thereby indicating that the aggregation validity of the questionnaire was appropriate. Third, MPLUS 8.3 was used to conduct confirmatory factor analysis (CFA). The fitting index of each model is shown in Table 3. The theoretical four-factor model (employee innovative behavior, coworker ostracism, leader innovation support, and workplace wellbeing) demonstrated a better fit with the data (χ2/df = 1.676, CFI = 0.947, TLI = 0.941, RMSEA = 0.046, and SRMR = 0.053) compared with the other models, thereby indicating that the theoretical four-factor model exhibited appropriate discriminant validity. Moreover, as shown in Table 4, the square root of the AVE of all the variables was larger than the correlation of all the remaining constructs in the rows and columns, thereby indicating that the discriminant validity of the questionnaire was appropriate.

**TABLE 2:** Reliability and validity of variables.

Variable	Load factor	Cronbach’s α	KMO	CR	AVE
EIB	0.646–0.776	0.780	0.811	0.846	0.478
CO	0.665–0.819	0.922	0.949	0.935	0.592
LSI	0.715–0.824	0.775	0.772	0.857	0.600
WWB	0.676–0.855	0.838	0.848	0.890	0.619

EIB, employee innovative behavior; CO, coworker ostracism; LSI, leader support for innovation; WWB, workplace wellbeing.

**TABLE 3:** Results of confirmatory factor analyses.

Models	χ^2^	df	χ^2^/df	Δχ^2^	CFI	TLI	RMSEA	SRMR
Four-factor model (EIB; CO; LSI; WWB)	450.800	269	1.676	/	0.947	0.941	0.046	0.053
Three-factor model (EIB + LSI; CO; WWB)	624.773	272	2.297	173.973	0.898	0.888	0.064	0.062
Two-factor model (EIB + WWB; CO + LSI)	1037.462	274	3.786	586.662	0.779	0.758	0.093	0.117
One-factor model (EIB + CO + LSI + WWB)	1937.589	275	7.046	1486.789	0.520	0.476	0.138	0.172

EIB, employee innovative behavior; CO, coworker ostracism; LSI, leader support for innovation; WWB, workplace wellbeing.

**TABLE 4:** Means, standard deviations (SDs), and correlations.

Variables	1	2	3	4	5	6	7	8	9
1. Gender	–								
2. Education	0.132*	–							
3. Age	–0.045	0.223**	–						
4. Department	0.148**	−0.189**	−0.351**	–					
5. Working seniority	0.021	0.152**	0.807**	−0.295**	–				
6. EIB	−0.168**	0.102	0.173**	−0.204**	0.143*	(0.692)			
7. CO	–0.107	−0.157**	−0.147**	0.146**	−0.128*	−0.125*	(0.769)		
8. LSI	–0.024	0.025	0.103	−0.146**	0.136*	0.416**	−0.193**	(0.775)	
9. WWB	–0.044	0.105	0.238**	−0.184**	0.161**	0.469**	–0.088	0.422**	(0.787)
M	1.50	2.92	2.08	2.59	1.94	4.06	1.96	3.91	3.53
SD	0.501	0.636	0.889	1.440	0.892	0.569	0.794	0.699	0.805

*N* = 319; *p < 0.05, **p < 0.01. Values in parentheses are square roots of AVE. EIB, employee innovative behavior; CO, coworker ostracism; LSI, leader support for innovation; WWB, workplace wellbeing.

### Common method variance

As all the variables in this study were measured *via* the employees’ self-evaluation, the problem of common method variance should be considered. Therefore, the Harman single-factor method was used for the testing, and unrotated principal component analysis was conducted for all the variables. The results showed that the first factor explained 22.96% of the cumulative total variance, which is less than 40% and meets the recommended criterion. Furthermore, CFA was conducted with the inclusion of the latent common factor model. The results revealed that the fitting effect of the latent common factor model (χ2/df = 1.734, CFI = 0.943, TLI = 0.936, RMSEA = 0.048, and SRMR = 0.065) was not as good as that of the four-factor model (χ2/df = 1.676, CFI = 0.947, TLI = 0.941, RMSEA = 0.046, and SRMR = 0.053), thereby indicating the absence of common method variance in this study.

### Descriptive statistics and correlation analysis

Table 4 presents the descriptive statistics (mean and SD) and correlation analysis (Pearson’s coefficient). The results showed that employee innovative behavior was positively correlated with workplace wellbeing (*r* = 0.469, *P* < 0.01) but negatively associated with coworker ostracism (*r* = −0.125, *P* < 0.05), and a non-significant correlation existed between coworker ostracism and workplace wellbeing (*r* = −0.088, *P* > 0.05). In addition, employee innovative behavior was positively associated with leader support for innovation (*r* = 0.416, *P* < 0.01), and leader support for innovation was positively associated with workplace wellbeing (*r* = 0.422, *P* < 0.01) but negatively associated with coworker ostracism (*r* = −0.193, *P* < 0.01). Among the results, the correlation between coworker ostracism and workplace wellbeing was unexpected. The hypothesis tests were further conducted.

### Hypothesis tests

First, SPSS 26.0 was used for the hierarchical regression analysis to verify the research hypotheses, and the results are shown in Table 5.

**TABLE 5:** Hierarchical regression analysis results.

Variables	LSI		CO		WWB			
	Model 1	Model 2	Model 3	Model 4	Model 5	Model 6	Model 7	Model 8
Gender	–0.015	0.075	−0.18*	−0.205*	–0.033	0.078	0.078	0.054
Education	–0.007	–0.041	–0.126	–0.117	0.053	0.011	0.011	0.024
Age	–0.046	–0.069	–0.072	–0.066	0.236**	0.207**	0.207**	0.229**
Department	−0.059*	–0.034	0.062	0.054	–0.06	–0.028	–0.028	–0.017
Working seniority	0.116	0.104	–0.011	–0.007	–0.078	–0.093	–0.093	–0.126
EIB		0.504***		–0.144		0.624***	0.624***	0.464***
CO							0.001	
LSI								0.317***
R^2^	0.032	0.187	0.058	0.068	0.073	0.252	0.252	0.314
ΔR^2^		0.155		0.01		0.179	0	0.061
F	2.069	59.684***	3.836**	3.293	4.937***	74.814***	0.001	27.820***

*N* = 319; *p < 0.05, **p < 0.01, ***p < 0.001. EIB, employee innovative behavior; CO, coworker ostracism; LSI, leader support for innovation; WWB, workplace wellbeing.

Table 5 reveals that employee innovative behavior was positively related to workplace wellbeing (β = 0.624, *P* < 0.01, model 6), thereby supporting Hypothesis 1. Compared with model 6, the mediating variable coworker ostracism was added to model 7, which showed no significant effect on workplace wellbeing (β = 0.001, *P* > 0.05, model 7). However, the influence coefficient of employee innovative behavior on workplace wellbeing did not change (β = 0.624, *P* < 0.01), thereby indicating that coworker ostracism did not play a mediating role in the relationship between employee innovative behavior and workplace wellbeing; thus, Hypothesis 2 was unsupported. Compared with model 6, the mediating variable leader support for innovation was added to model 8, which demonstrated that leader support for innovation was positively related to workplace wellbeing (β = 0.317, *P* < 0.01). However, the influence coefficient of employee innovative behavior on workplace wellbeing decreased significantly (β = 0.464, *P* < 0.01), indicating that leader support for innovation played a partial mediating role in the relationship between employee innovative behavior and workplace wellbeing; thus, Hypothesis 3 was supported.

Second, PROCESS macro was employed for the bootstrap analysis. The sample size was set to 5,000, and the confidence interval was set to 95%. The non-parametric percentile method of deviation correction was selected for the bootstrap sampling, and the results are presented in Table 6. The figure shows that the direct effect of employee innovative behavior on workplace wellbeing was 0.466, and its 95% CI was [0.318, 0.615] (excluding 0), thereby further supporting Hypothesis 1. In addition, the indirect effect of employee innovative behavior on workplace wellbeing through coworker ostracism was−0.002, with a 95% CI of [−0.019, 0.015] (including 0). Thus, the mediating effect of coworker ostracism was not confirmed, and Hypothesis 2 was unsupported. The indirect effect of employee innovative behavior on workplace wellbeing through leader support for innovation was 0.163, and the 95% CI was [0.084, 0.265] (excluding 0). Therefore, the mediating effect of leader support for innovation was confirmed, and Hypothesis 3 was further supported. The chain mediation path effect value of “employee innovative behavior → leader support for innovation → coworker ostracism → workplace wellbeing” was−0.004, and the CI was [−0.016, 0.007] (including 0). Therefore, Hypothesis 4 was unsupported.

**TABLE 6:** Results of the chain mediation test.

Path	Effect	S.E	95% CI	
			Lower limit	Upper limit
EIB→WWB	0.466	0.076	0.318	0.615
EIB→CO→WWB	–0.002	0.008	–0.019	0.015
EIB→LSI→WWB	0.163	0.046	0.084	0.265
EIB→LSI→CO→WWB	–0.004	0.006	–0.016	0.007

EIB, employee innovative behavior; CO, coworker ostracism; LSI, leader support for innovation; WWB, workplace wellbeing.

**FIGURE 2:**
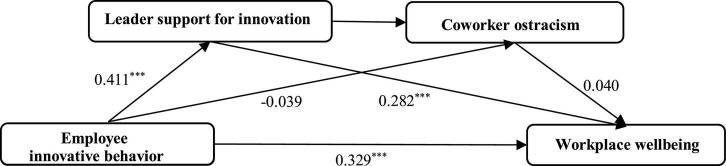
Results of the theoretical model using MPLUS. *N* = 319, ***p < 0.001. Standardized path coefficients are reported.

In addition, to validate the model more completely, MPLUS 8.3 is used to construct a structural equation model. Figure 2 presents the standardized path coefficients of the model. In Figure 2, the direct effect of employee innovative behavior on workplace wellbeing is supported by the regression coefficient and associated significance level (β = 0.329, *p* < 0.001). Furthermore, in Table 7, the total effect coefficient of employee innovative behavior on workplace wellbeing is significant (β = 0.623, *p* < 0.001), and the 95% CI is [0.465, 0.775] (excluding 0). The results suggest that employee innovative behavior is significantly positively related to workplace wellbeing. Thus, Hypothesis 1 is confirmed. Second, in Figure 2, employee innovative behavior has no significant effect on coworker ostracism (β =−0.039, *p* > 0.05), and coworker ostracism has no significant effect on workplace wellbeing (β = 0.040, *p* > 0.05). Furthermore, in Table 7, after controlling leader support for innovation, the indirect effect of “EIB→CO→ WWB” is not significant (β =−0.002, *p* > 0.05), and the CI is [−0.027, 0.007] (including 0). The results suggest that the mediating effect of coworker ostracism between employee innovative behavior and workplace wellbeing is not supported. Thus, Hypothesis 2 is not confirmed. Third, Figure 2 shows that employee innovative behavior is positively related to leader support for innovation (β = 0.411, *p* < 0.001), and leader support for innovation is positively related to workplace wellbeing (β = 0.282, *p* < 0.001). Furthermore, in Table 7, after controlling coworker ostracism, the indirect effect of “EIB→LSI→WWB” is significant (β = 0.164, *p* < 0.001), and the CI is [0.085, 0.271] (excluding 0). The results suggest that leader support for innovation plays a mediating role between employee innovative behavior and workplace wellbeing. Thus, Hypothesis 3 is confirmed. Finally, in Figure 2, employee innovative behavior had a positive effect on leader support for innovation (β = 0.411, *p* < 0.001), leader support for innovation exhibited a direct positive effect on coworker ostracism (β =−0.157, *p* < 0.05), but coworker ostracism has no significant effect on workplace wellbeing (β = 0.040, *p* > 0.05). The indirect impact of employee innovative behavior on workplace wellbeing by means of two chain-mediating variables, leader support for innovation and coworker ostracism, was unsupported. Furthermore, in Table 7, the indirect effect of “EIB→LSI→CO→WWB” is not significant (β =−0.004, *p* > 0.05), and the CI is [−0.019, 0.006] (including 0). The results suggest that the chain-mediating effect of “leader support for innovation–coworker ostracism” between employee innovative behavior and workplace wellbeing is not supported. Thus, Hypothesis 4 is not confirmed.

**TABLE 7:** Results of multiple mediating effect test.

Effects	Estimate	S.E	*P*	95% CI	
				Lower limit	Upper limit
Total effect EIB→WWB	0.623	0.081	0.000	0.465	0.775
Direct effect EIB→WWB	0.329	0.051	0.000	0.226	0.428
Direct effect EIB→CO	–0.039	0.073	0.591	–0.187	0.095
Direct effect CO→WWB	0.040	0.055	0.469	–0.082	0.136
Direct effect EIB→LSI	0.411	0.074	0.000	0.249	0.544
Direct effect LSI→WWB	0.282	0.061	0.000	0.152	0.391
Direct effect LSI→CO	–0.157	0.066	0.017	–0.283	–0.028
Indirect effect EIB→CO→WWB	–0.002	0.008	0.777	–0.027	0.007
Indirect effect EIB→LSI→WWB	0.164	0.047	0.000	0.085	0.271
Indirect effect EIB→LSI→CO→WWB	–0.004	0.006	0.536	–0.019	0.006

EIB, employee innovative behavior; CO, coworker ostracism; LSI, leader support for innovation; WWB, workplace wellbeing.

## Discussion

Based on social comparison theory and social exchange theory, this study introduces coworker ostracism and leader support for innovation as mediating variables to explore the bright side and dark side of the effect of employee innovative behavior on employees’ workplace wellbeing. The empirical findings are described below.

First, the results show that employee innovative behavior is positively and directly related to workplace wellbeing. As employee innovative behavior is beneficial to enterprises’ development, employees who engage in innovation will perceive self-goal satisfaction and self-value realization, which can improve their workplace wellbeing.

Second, coworker ostracism does not negatively mediate the relationship between employee innovative behavior and workplace wellbeing, employee innovative behavior is negatively correlated with coworker ostracism, and no significant correlation exists between coworker ostracism and workplace wellbeing. This finding may be attributed to the following reasons. First, most of the survey participants reported that they have not been ostracized by their coworkers, perhaps because the perceived coworker ostracism scale reported by the employees cannot accurately reflect actual coworker ostracism. Moreover, the items in the coworker ostracism scale developed by Ferris et al. (2008) describe coworker ostracism directly. However, in reality, coworker ostracism has the characteristic of concealment. Second, the popularity of team cooperation in enterprises makes the interests of employees and coworkers closely related. Thus, employees tend to try their best to maintain the harmony.

Third, employee innovative behavior indirectly affects workplace wellbeing through leader support for innovation. Innovation consistently benefits the development of enterprises; thus, employees will receive innovative support from leaders in the process of engaging in innovative behavior. Specifically, when employees engage in innovative behavior, their leader will provide resource support, encouragement, and praise, which can lead to high-quality leader–member exchange and enhance their workplace wellbeing.

Finally, the chain-mediating effect of leader support for innovation and coworker ostracism on the relationship between employee innovative behavior and workplace wellbeing is unverified, but the negative correlation between leader support for innovation and coworker ostracism is significant. The absence of the chain-mediating effect may also be attributed to the “hidden” phenomenon of coworker ostracism.

### Theoretical implications

First, a new perspective is provided in this study by taking employee innovative behavior as an antecedent to explore the subsequent influence path at the individual level, thereby expanding research on employee innovative behavior as an antecedent. Previous studies on employee innovative behavior consistently regarded such behavior as an outcome variable and discussed the antecedents that may lead to employee innovative behavior from the perspective of the organizational level, individual level, and task characteristics (Janssen, 2000; Wu et al., 2011; Kang et al., 2016; Tian et al., 2020; Kim et al., 2021; Wang Y. et al., 2021; Elsetouhi et al., 2022). However, little attention was paid to employees’ innovative behavior as an antecedent, and only few studies discussed the positive influence of employees’ innovative behavior on organizational performance (Laforet, 2011; Aryee et al., 2012). In addition, some studies have started to focus on the dark side of employees’ innovative behavior in recent years (Hammond et al., 2019; Ng and Wang, 2019; Nguyen and Le, 2019; Breidenthal et al., 2020; Dadaboyev et al., 2021), but the relationship between innovative behavior and employee wellbeing was ignored. Only one study presented a conceptual model of how to moderate the negative effects of employee creativity on wellbeing (Mustafa and Ramos, 2018). More importantly, to our knowledge, no research integrates the double-edged sword effect of employee innovative behavior on workplace wellbeing. This study takes employee innovative behavior as an antecedent and explores both the positive and negative effects of employee innovative behavior on workplace wellbeing, which will enrich the research on employee innovative behavior.

Second, based on social comparison theory and social exchange theory, coworker ostracism and leader support for innovation are introduced in this study as two mediating variables to reveal how employee innovative behavior affects workplace wellbeing. Although some studies presented the dark side of employee creativity (Janssen, 2003; González-Romá and Hernández, 2016; Ng and Wang, 2019; Breidenthal et al., 2020), few studies on employee innovative behavior considered coworkers’ attitude and leaders’ attitude toward employees’ innovative behavior. As research showed that the role of coworkers and leaders cannot be ignored when exploring the outcomes of innovation, as they always play a crucial role in the process of employees’ innovative behavior (Chiaburu and Harrison, 2008; Sijbom et al., 2015a,b). This study explored the double-edged sword effect of employee innovative behavior on workplace wellbeing using coworker ostracism and leader support for innovation as mediating variables. In addition, the chain-mediating effect of leader support for innovation and coworker ostracism is explored in this study, and the effect of leaders as a power distributor on coworker ostracism is examined. Thus, the current study enriches relevant research on the relationship between employee innovative behavior and workplace wellbeing.

Third, the mediating effect of coworker ostracism between employee innovative behavior and workplace wellbeing was unsupported in this study. This finding may be due to the strong “concealment” of coworker ostracism in the context of Chinese collectivist culture. In fact, coworker ostracism is an anti-regulatory behavior involving ambiguous and low-intensity individual intentions that is difficult to identify compared to other interpersonal maltreatment such as bullying and aggression (Ferris et al., 2017; Naseer et al., 2018). Especially in the context of Chinese Confucian culture, which emphasizes that “harmony is the most valuable,” people generally repress their grievances instead of expressing them directly to others. Therefore, coworker ostracism may manifest in implicit and imperceptible ways.

### Practical implications

In this era, when innovation has become a general trend, enterprises should pay attention to follow-up support for employees’ innovative behavior and avoid discouraging their enthusiasm for innovation to enhance the innovation vitality of the enterprise and realize sustainable development.

First, enterprises should pay attention to the outstanding innovation performance of their employees. Innovation is the key to the core competitiveness of an enterprise. When employees engage in innovative behavior, leaders should give them innovation support as much as possible in terms of both innovation resources and emotional encouragement, which is not only conducive to improving employees’ workplace wellbeing but also beneficial to promote organizational development.

Second, enterprises should help their employees overcome the obstacles they may encounter in the process of innovation to avoid the tragedy of “dying on the way” to innovation. Innovation, which means change or breakthrough, may threaten the interests of certain individuals in the organization and thus is hindered. Therefore, when employees actively explore and strive for innovation, leaders should support and encourage them.

Finally, enterprises should create a harmonious atmosphere of organizational innovation and teamwork. A harmonious working atmosphere has become an important factor in attracting job seekers and retaining employees. Therefore, enterprises should adopt measures to create a harmonious atmosphere to avoid coworker ostracism in the workplace.

### Limitations and future research

First, a two-wave design in the survey was used to reduce CMV in this study. However, all variables came from a single source and were employee self-reported, which limits the conclusions that can be made regarding causality. Thus, multiple resources can be adopted to reduce the threat of a CMV in subsequent research. Researchers can collect the data from multiple sources. For example, employees evaluate their perceived workplace wellbeing, coworker ostracism, and leader support for innovation, whereas leaders evaluate their innovative behavior.

Second, coworker ostracism in this study was measured with the scale developed by Ferris et al. (2008), which defines coworker ostracism as the subjective feeling of being ignored, avoided, or excluded by coworkers in the workplace. Given that coworker ostracism manifests in implicit and imperceptible ways. The coworker ostracism scale should be developed further for future studies.

Third, to thoroughly explore the reactions of coworkers and leaders to employee innovative behaviors, a qualitative investigation can be chosen in future research, including interviewing employees or using a recall paradigm. These methods could be used to ask participants to describe recent incidents in which they were rejected by coworkers and supported by leaders for innovation at work. Furthermore, to avoid the limitations of memory distortion and recall bias, details of incidents of coworker ostracism and leadership innovative support could be collected qualitatively or quantitatively in real time using experience sampling methods.

Lastly, this study chooses coworker ostracism and leader support for innovation as mediating variables. Future research can consider other mediators, such as work alienation and repercussions. Work alienation is a negatively dissociate state of the individual concerning the product or process of work, coworker jealousy and disconnection triggered by employees’ innovative behavior may lead to alienation from a person’s job (Shantz et al., 2015). However, employees may cope with alienation by being “innovative” so that they can create situations at work that are meaningful to them (Mitchell, 1984). Therefore, it would be meaningful for future research to clarify the mixed effects involved. Moreover, although the results show that employee innovative behavior has a positive influence on leader support for innovation, future research could investigate the acceptance of different leadership orientations (mastery orientation vs. performance orientation) on employee innovative behavior. In addition, future research may consider the effects of moderating variables, such as innovative style, organizational context (Janssen et al., 2004), task interdependence (Dadaboyev et al., 2021), and LMX (Nelson, 2017; Breidenthal et al., 2020). For instance, employees with a high-quality LMX relationship may be more ostracized by their coworkers and be more supported by their leaders. Moreover, individual characteristics should be considered, like extraversion, agreeableness, or conscientiousness (Howard et al., 2020).

## Data availability statement

The raw data supporting the conclusions of this article will be made available by the authors, without undue reservation.

## Ethics statement

As a protection of all participants, all subjects read informed consent before participating in this study and voluntarily made their decision to complete surveys. The protocol was approved by an Institutional Review Board at Xiangtan University of China.

## Author contributions

XC and MX: investigation. XC: data analysis and writing—original draft preparation. HW (1st author): supervision and writing—review and editing. HW (3rd author): writing—revising manuscript. All authors read and agreed to the published version of the manuscript.
